# Comparison of Orthodontic Tooth Movement of Regenerated Bone Induced by Carbonated Hydroxyapatite or Deproteinized Bovine Bone Mineral in Beagle Dogs

**DOI:** 10.3390/ma17010112

**Published:** 2023-12-25

**Authors:** Takaharu Abe, Ryo Kunimatsu, Kotaro Tanimoto

**Affiliations:** 1 Department of Orthodontics, Division of Oral Health and Development, Hiroshima University Hospital, Hiroshima 734-8553, Japan; takabe@hiroshima-u.ac.jp; 2 Department of Orthodontics and Craniofacial Developmental Biology, Hiroshima University Graduate School of Biomedical & Health Sciences, Hiroshima 734-8553, Japan; tkotaro@hiroshima-u.ac.jp

**Keywords:** bone regeneration, carbonated hydroxyapatite, deproteinized bovine bone mineral, orthodontic tooth movement

## Abstract

Orthodontic treatments often involve tooth movement to improve dental alignment. In this study, we aimed to compare tooth movement in regenerated bone induced by two different bone fillers, carbonated hydroxyapatite (CAP) and deproteinized bovine bone mineral (DBBM). Four beagle dogs were used in this comparative study. The first, second, and fourth lower mandibular premolars (P1, P2, and P4) on both sides of the mouth were extracted, and CAP was implanted into the extraction site on the left side and DBBM into the right side. Following regenerative bone healing, orthodontic devices were attached to perform orthodontic tooth movement of the lower third mandibular premolar (P3) on both sides. X-ray examination, intraoral scan, and histological analysis were performed. The Mann–Whitney U test was used for statistical analysis, and *p* < 0.05 was considered significant. Bone regeneration and orthodontic tooth movement were observed in the CAP and DBBM groups. Histologically, normal periodontal tissue remodeling was observed on the compression and tension sides of CAP and DBBM. No statistical difference was observed in the number of osteoclasts around the periodontal ligament and the root resorption area. Orthodontic tooth movement of regenerated bone induced by CAP and DBBM was therefore achieved.

## 1. Introduction

Various bone filling materials, such as collagen gel [1], deproteinized bovine bone mineral (DBBM) [2], hydroxyapatite (HA) [3], and β-tricalcium phosphate (β-TCP) [4], have been used for alveolar bone regeneration and dental treatment.

For orthodontic purposes, bone filler is typically transformed into physiological bone after it is grafted into the body, serving its intended metabolic purpose. In contrast to bone augmentation in implant therapy, a bone filler placed into a tooth socket (followed by orthodontic treatment) must be bioabsorbable to allow tooth movement.

Of the bone filler candidates, collagen exhibits disadvantages such as low physical strength and difficulty maintaining its shape within the oral cavity. Similarly, HA does not allow orthodontic tooth movement at the implantation site because it cannot be replaced by bone in the long term [5]. Furthermore, β-TCP is hydrolyzed and dissolved in body fluids, resulting in bone regeneration while maintaining its original shape and density [6].

Jensen et al. [2] reported that DBBM implantation can effectively regenerate bones. DBBM was approved in Japan in 1999 and is widely used as a bone augmentation material for implant placement [7]. Studies indicate that DBBM will eventually be replaced by natural bone over time [8,9]. Araújo et al. [10] reported that DBBM did not interfere with tooth movement if it was implanted in the tooth extraction site and orthodontic force was applied.

Carbonated hydroxyapatite (CAP) is another artificial biomaterial that was approved for clinical use in Japan in 2017 [11]. CAP contains 6–9% carbonate in its apatite structure and exhibits high osteoconductivity and remodeling ability [11]. Mano et al. [12] observed alveolar ridge continuity after CAP implantation. Fukuda et al. [13] demonstrated that CAP was effective in maintaining the trabecular bone after tooth extraction. Therefore, owing to these favorable properties, CAP is frequently used in dental treatment. CAP is currently being applied clinically as a bone filler for bone augmentation and regeneration of periodontal tissue in implant-bearing regions [14,15]. 

It has been previously reported that regenerated bone induced by CAP and mesenchymal stem cells (MSCs) can produce orthodontic tooth movement [16]. However, no studies have investigated tooth movement in regenerated bone using CAP without MSCs and compared it with other bone filler materials. Therefore, in this study, we aimed to evaluate the effects of DBBM and CAP as bone filling materials on bone regeneration and tooth movement.

## 2. Materials and Methods

This study was approved by the relevant ethics review board of Hamley Co., Ltd. (Ibaraki, Japan) Animal Ethics Committee [Study No. 20-H022].

### 2.1. Scaffold Implantation

Four dogs (TOYO beagles, 12 months old; Kitayama Labes, Nagano, Japan) were anesthetized via intramuscular injection with 0.4 mL/kg of ketamine hydrochloride (Ketalal 500 mg, Daiichi Sankyo Propharma, Tokyo, Japan) and xylazine (Theratal 2% injection, Bayer Yakuhin, Osaka, Japan) in a 1:1 mixed solution. The same solution was also administered to maintain anesthesia. 

The operation area was sterilized with an Isodine solution for animals (Mundipharma Co., Ltd., Tokyo, Japan; 20 mg of Japanese Pharmacopoeia povidone-iodine in 1 mL), and xylocaine was used to induce local anesthesia. The first, second, and fourth premolars (P1, P2, and P4) of the mandible were then extracted. CAP obtained from Cytrans Granules^®^ (GC, Tokyo, Japan) and DBBM obtained from Bio-Oss^®^ (Geistlich Pharma AG, Wolhusen, Switzerland) were used for the experiments. The bone filling material was implanted in the extraction sites and the gingiva was sutured to close the wound. DBBM S (0.25–1.0 mm in size) was implanted on the right side, and CAP S (0.3–0.6 mm) and M (0.6–1.0 mm) mixed at a 1:1 ratio were implanted on the left side. Healing progress was checked monthly for 3 months after Isodine disinfection.

### 2.2. Attaching Orthodontic Appliances and Moving Teeth

Healing of the extraction site was confirmed 6 weeks after transplantation, and an impression was taken using silicone material (Examixfine Regular type; GC) and a tray. Three months after implantation, an orthodontic appliance manufactured by Wada Seimitsu Laboratory using a casting method was attached. We used a stainless steel wire measuring 0.019 × 0.025 inches for the appliance, and for the crown on P3, we used 0.022-inch slotted tubing. Subsequently, the closed coil spring (TOMY International, Tokyo, Japan) was adjusted to 100 g, and experimental tooth movement was performed. The date of device attachment was defined as day 0, and observations were conducted every 2 months.

### 2.3. Intraoral and Radiographic Photographs

After tooth extraction and transplantation, radiographs were taken every month for 3 months to confirm healing and bone regeneration. Dental radiographs and intraoral photographs were taken at the start of the orthodontic movement and every 2 months thereafter. Intraoral photographs were captured in the same direction.

### 2.4. Evaluation of Tooth Movement by an Intraoral Scanner (IOS)

**Figure 1:**
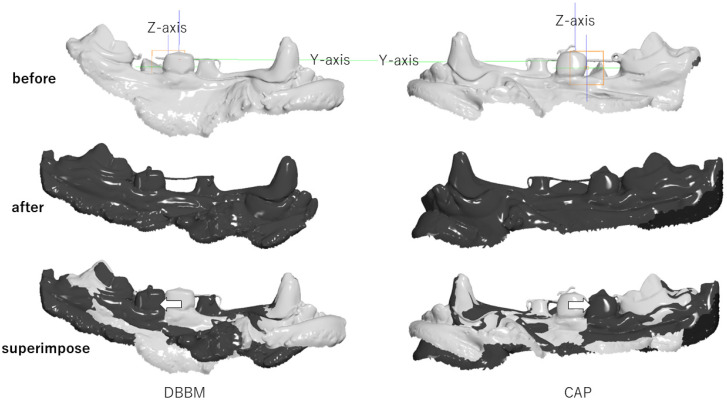
The STL data were superimposed, mainly for the canine (C) and first molar (M1). The Z-axis was the center of P3 with the device attached. The Y-axis was the direction of the wire. The blue line indicates the Z-axis, the green line indicates the Y-axis, and the orange square line indicates the X-axis direction. The white arrow indicates the direction of movement of P3.

Tooth movement was recorded at the start of the movement and every 2 months after using an intraoral scanner (IOS) (Adva IOS 100, GC, Tokyo, Japan), and Viewbox 4 (version 4.1.0.12) (dHAL Software, Kifissia, Greece) was used to assess tooth movement. The IOS was focused on the canine (C) and first molar (M1): the Z-axis was the center of P3 when the device was attached; the Y-axis was the direction of the wire; and the X-axis followed the occlusal plane from the intersection of the Y and Z axes. Using these planes, the movement distance and inclination angle of the teeth were observed and recorded. Figure 1 depicts the three-dimensional (3D) superimposition of the IOS images before and after tooth movement using Viewbox 4. It should be noted that the evaluation of tooth movement was excluded from the results of this study because a large tilt of the crown was observed in one dog due to a wire fracture. 

### 2.5. Histological Evaluation of Regenerated Bone

At the end of the movement, an excessive amount of thiamylal sodium (Isososol, Nichi-Iko, Tokyo, Japan) was intravenously administered (50 mg/kg or more) to euthanize the dogs. Excess bone, teeth, and soft tissue were removed after harvesting. Tissues were fixed with 4% Paraformaldehyde (PFA), decalcified with ethylenediaminetetraacetic acid (EDTA) for 1 month. Sections were stained with hematoxylin and eosin (H and E), Masson’s trichrome (MT), and tartrate-resistant acid phosphatase (TRAP) after being sliced into 5-μm thick slices. The sagittal section was cut between the P3 and the first posterior molar. The number of TRAP-positive cells per unit area of alveolar bone on the compression side of the moved tooth was counted. For H and E staining, the tooth root resorption area on the compression side of the moved tooth was measured using ImageJ software https://imagej.net/ij/download.html (accessed 22 December 2023) (National Institute of Health, Bethesda, MD, USA).

### 2.6. Statistical Analysis

Results are presented as the mean ± standard deviation (SD). The Mann–Whitney U test was used for statistical analysis and *p* < 0.05 was considered significant. 

## 3. Results

### 3.1. Intraoral and Radiographic Evaluation

**Figure 2:**
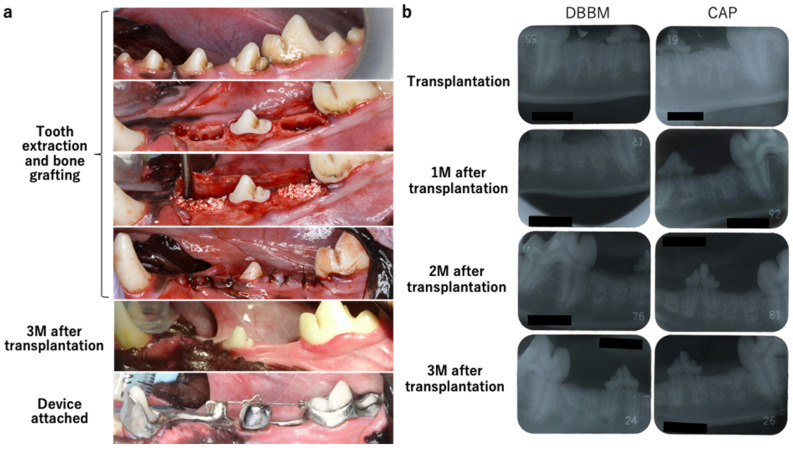

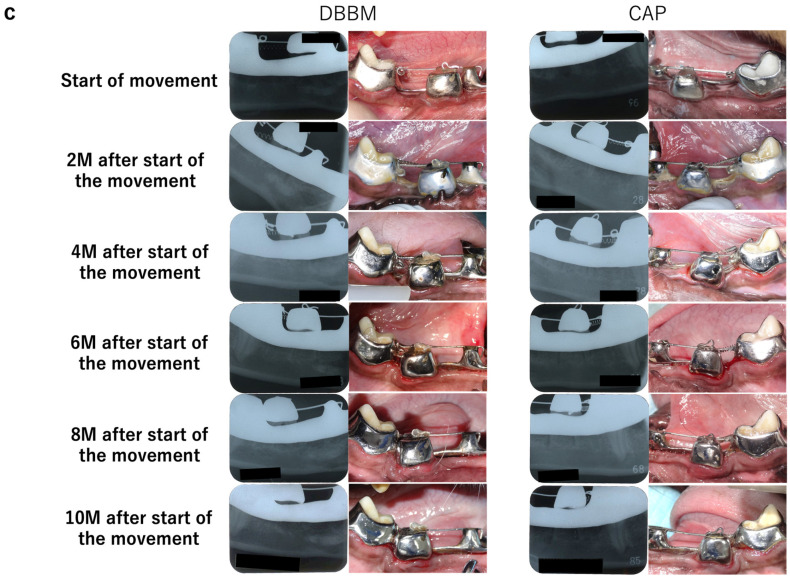
Intraoral photographs and dental radiographs obtained during tooth extraction and bone filler grafting. (**a**) Intraoral photograph. Tooth extraction and carrier implantation were performed. The state of healing 3 months after transplantation and a photograph of the attached device are shown. (**b**) Dental radiographs at the time of transplantation and 1-, 2-, and 3 months post-transplantation. DBBM was implanted on the right side, and CAP was implanted on the left side. Two months after the transplantation, trabecular formation was observed. (**c**) Intraoral and dental X-ray photographs every 2 months after the movement started.

Intraoral photographs and dental radiographs obtained during tooth extraction and bone filler grafting are shown in Figure 2a,b. One month after implantation, the implanted bone filler was replaced with bone in both groups; trabecular bone formation was observed 2 months after implantation. Intraoral photographs and dental X-ray images captured during the orthodontic tooth movement every 2 months are shown in Figure 2c. 

### 3.2. 3D Tooth Movement Evaluation

**Figure 3:**
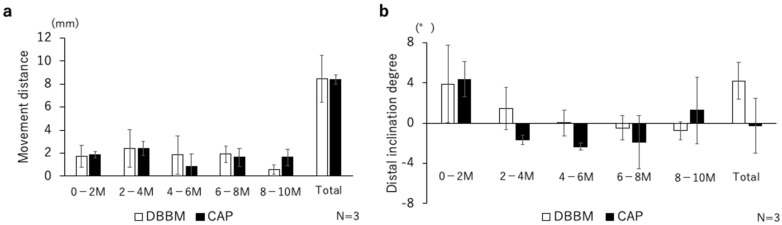
(**a**) Migration distance of P3 along the Y-axis during centrifugation. No significant differences were observed for either schedule. (**b**) Rotation angle toward centrifugation around the X-axis. DBBM tended to have a larger centrifugal slope than CAP but no significant differences were observed. The total movement and inclination indicate the changes between 0 M and 10 M.

The evaluation of the tooth movement distance and inclination are shown in Figure 3a,b. There were no marked differences between the DBBM and CAP groups in terms of tooth movement distance. At the total distal inclination degree, the distal tooth inclination in the DBBM group tended to be higher than that in the CAP group. 

### 3.3. Histological Evaluation

**Figure 4:**
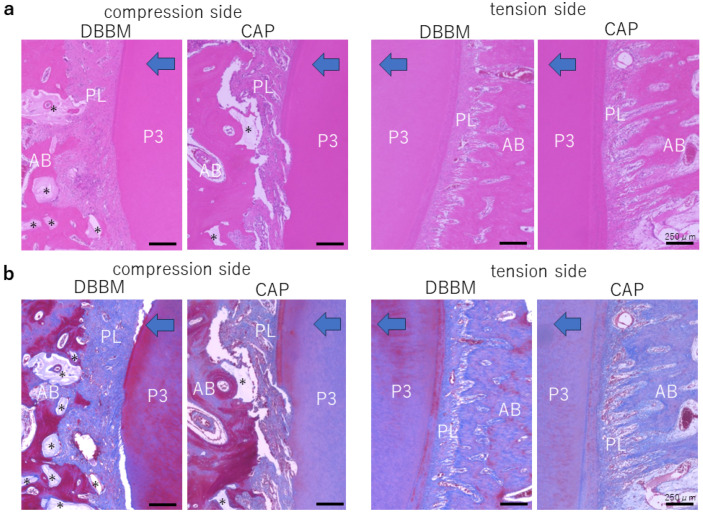
(**a**) H and E staining. On the compressed side, resorption was observed in part of the alveolar bone, cementum, and dentin. On the tension side, bone formation was observed. (**b**) MT staining. M1 teeth are shown. The bone is formed in layers around the bone filler. The newly formed bone is stained blue on the traction side. The blue arrow indicates the direction of tooth traction. * indicates bone filler, P3 indicates the third premolar AB indicates alveolar bone, and PL indicates periodontal ligament; scale bar = 250 μm.

H&E and MT staining images on the compression and traction sides are shown in Figure 4a,b. On the compressed side, the periodontal ligament fibers were disturbed; resorption was observed in part of the alveolar bone, cementum, and dentin. On the tension side, periodontal ligament fibers were elongated, blood vessel permeability was enhanced, and bone formation was observed perpendicular to the long axis of the tooth root. Following MT staining, the periodontal ligament was stained blue, and layers of bone formation were observed around the bone filler. Newly formed bone was stained blue on the traction side.

**Figure 5:**
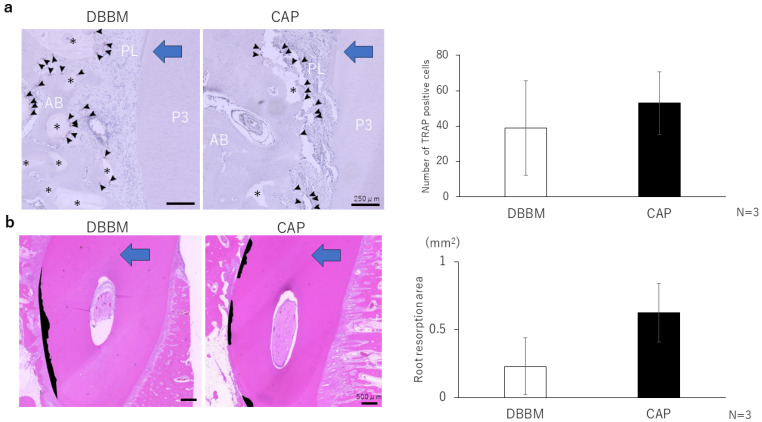
(**a**) TRAP staining. P3 teeth are shown. The arrowheads indicate TRAP-positive osteoclasts. Osteoclasts were observed around the alveolar bone on the pressing side and around the bone filler. No marked difference was observed in TRAP-positive cells. * indicates bone filler, P3 indicates the third premolar AB indicates alveolar bone, and PL indicates periodontal ligament; Scale bar = 250 μm. (**b**) Black areas indicate resorbed roots. No significant difference was observed in the root resorption area. Scale bar = 500 μm. The blue arrow indicates the direction of tooth traction.

Most of TRAP-positive cells were observed around the alveolar bone on the compression side of the moved tooth (Figure 5a). The number of TRAP-positive cells observed around the compressed periodontal ligament in the DBBM and CAP groups was 39.0 ± 26.6 and 53.0 ± 17.7, respectively. The root resorption area was 0.23 ± 0.21 mm^2^ in DBBM and 0.62 ± 0.22 mm^2^ in CAP. CAP tended to have a larger area resorption, but there was no statistical difference between the two groups (Figure 5b). 

## 4. Discussion

In this study, a bone substitute was implanted into the tooth extraction site, and orthodontic tooth movement was performed. Filling a tooth extraction site with bone filler has been clinically applied for socket preservation, and its effectiveness has been demonstrated [17]. Numerous reports have documented the application of bone filler implants for bone regeneration [18]. In this study, orthodontic tooth movement was performed on regenerated bone. 

It has been suggested that the carbonate content of bone filler is one of the factors contributing to its enhanced osteoconductivity [19]. Furthermore, CAP is stable in vivo and is rapidly absorbed in the weakly acidic environment (pH 5.3) of osteoclast Howship’s lacunae. The absorption of carriers by osteoclasts sprouts calcium and carbonate ions, and the increase in extracellular calcium ion concentrations promotes mineralization of the matrix by osteoblasts [20]. Sato et al. [21] conducted a study in which CAP, β-TCP, HA, and DBBM were implanted into a three-walled bone defect and reported that CAP implantation resulted in the rapid recruitment of osteoclasts and endothelial cells. The rapid induction of bone regeneration by the early migration of osteoclasts is an important requirement for graft carriers.

Two months after transplantation, resorption of bone fillers and formation of trabecular bone due to bone regeneration were observed in both the CAP and DBBM groups via X-ray examination. Regarding tooth movement and root resorption, only a few granules were retained, and a complete replacement with bone was expected. However, resorption is caused by osteoclasts generated by tooth movement. Ishikawa et al. [11] reported that CAP was more susceptible to osteoclast resorption than DBBM, but the difference was not significant. 

When tooth movement was evaluated using a 3D model, no differences were observed in the distance of tooth movement between the DBBM and CAP groups. Orthodontic tooth movement is achieved by bone metabolism of the alveolar bone in the periodontium, and usually achieved clinically by inclining the crown toward the tooth movement. Subsequently, bone resorption of the alveolar bone at the root of the teeth occurs, leading to migration of the root. However, the mode of tooth movement may vary depending on the environment in the body and the placement status of the bracket. Specifically, depending on bone turnover of the alveolar bone, there may be cases in which the translation is beautiful angle without an inclination occurring, or the roots may be moved first, depending on the angle of the bracket. In this study, we used an SS wire with highly rigid properties, which is compatible with individual teeth, and an engineered device for experimental tooth movement. Angular wires with a size of 0.019 × 0.025 inches, which is large in clinical terms, and a real multibracket device were used to ensure as little inclination as possible. However, it is known that even hard wires can bend [22]. In both groups, the angle of tilt was up to four degrees, and the incidence of tilt was minimal, which is within the clinically possible extent of tooth movement.

Both bone fillers exhibited high biocompatibility, and a prolonged time period was required for complete bone replacement. Regarding the remaining bone filler, it is possible that osteoclasts on the pressure side absorbed the bone filler during orthodontic tooth movement and induced bone remodeling. However, by implanting bone filler and integrating orthodontic tooth movement, bone formation occurred while maintaining the alveolar bone level. Therefore, it is important to use correctly remodeled bone filler without impeding tooth movement or causing excessive root resorption by osteoclasts. It is known that root resorption occurs in more than 90% of cases as teeth move [23]. There are limited studies on the histological evaluation of the metabolism of bone grafting materials when teeth are moved after implantation. In this study, the bone fillers remained on the compression side and were remodeled by osteoclasts simultaneously with the alveolar bone. Araújo et al. [10] reported that the decomposition rate of bone filler increased on the compression side. Ma et al. [24] similarly reported that orthodontic force promoted the decomposition of bone substitute material, and that it was most effective after 4 weeks. Alalola et al. [25] reported that residual bone grafting materials restrict tooth movement and cause tooth root resorption as a side effect. However, most of the studies have focused on DBBM and β-TCP, and there are few reports on tooth movement using CAP. In the present study, a trend towards a larger root resorption area was observed in CAP compared to DBBM. As reported by Nan et al. [26], increased bone density at the graft site may have caused root resorption. However, as stated in the limitations, our results were not significantly different, and due to the small sample size, further research on root resorption is needed with respect to CAP transplantation. 

To improve bone formation, the application of MSCs with CAP can be used to enhance bone regeneration [27]. Suzuki et al. [28] reported that the use of immature osteoblasts in combination with a 3D scaffold resulted in good bone regeneration. Furthermore, when combined with FGF-2, CAP has shown promising results for treating periodontal tissues [15]. As previously stated [1,29,30] (Putranti et al., 2022; Hiraki et al., 2020b; Nakajima et al., 2018), in the future, greater precision of bone regeneration is expected, especially with the use of stem cells and their supernatant as a transplant carrier. In this study, we demonstrated that bone regeneration and tooth movement is possible by implanting CAP and DBBM into a tooth extraction site. An inherent limitation of this study’s design was the small sample size. We investigated dogs in this study to evaluate the scaffold through experimental tooth movement in larger animals that are more similar to humans. In accordance with Russell and Burch’s 3R principle, we used four dogs (*n* = 4). Moreover, major damage to the device occurred, so that one animal dropped out of the study. We performed our experiments under conditions as identical as possible, but there were individual differences in the actual movement of teeth in vivo due to metabolic differences. Therefore, the explanation of our study may have been confounded by the small sample size. Although CAP and DBBM are approved bone grafting materials for implantation in the human body [2,11], insufficient knowledge has been obtained on orthodontic tooth movement. The demonstration that tooth movement can be achieved in CAP may open further prospects for bone augmentation in patients with cleft lip/palate and bone defects. Future research on tooth movement models in humans should be developed through specific clinical studies.

## 5. Conclusions

Carbonated hydroxyapatite (CAP) and deproteinized bovine bone mineral (DBBM) were implanted into empty tooth sockets, and bone regeneration was observed; orthodontic tooth movement was initiated and observed. No significant difference was observed between the movement speed of the teeth and the number of osteoclasts induced on the compression side and root resorption area. These findings demonstrate that tooth movement is possible following bone regeneration using CAP and DBBM.

## Data Availability

The datasets used and/or analyzed during the current study are available from the corresponding author on reasonable request.
